# Enhanced Release of Calcium Ions from Hydroxyapatite Nanoparticles with an Increase in Their Specific Surface Area

**DOI:** 10.3390/ma16196397

**Published:** 2023-09-25

**Authors:** Urszula Szałaj, Agnieszka Chodara, Stanisław Gierlotka, Jacek Wojnarowicz, Witold Łojkowski

**Affiliations:** 1 Laboratory of Nanostructures, Institute of High Pressure Physics, Polish Academy of Science, Sokolowska 29/37, 01-142 Warsaw, Poland; xray@unipress.waw.pl (S.G.); j.wojnarowicz@labnano.pl (J.W.); w.lojkowski@labnano.pl (W.Ł.); 2 Faculty of Materials Engineering, Warsaw University of Technology, Wołoska 41, 02-507 Warsaw, Poland; 3 Leyton Poland Ltd., Wspólna 70, 00-687 Warsw, Poland; chodara.agnieszka@gmail.com

**Keywords:** hydroxyapatite (HAP), nanoparticles, microwave hydrothermal synthesis, calcium-ion release, solubility, size effect, specific surface area

## Abstract

Synthetic calcium phosphates, e.g., hydroxyapatite (HAP) and tricalcium phosphate (TCP), are the most commonly used bone-graft materials due to their high chemical similarity to the natural hydroxyapatite—the inorganic component of bones. Calcium in the form of a free ion or bound complexes plays a key role in many biological functions, including bone regeneration. This paper explores the possibility of increasing the Ca^2+^-ion release from HAP nanoparticles (NPs) by reducing their size. Hydroxyapatite nanoparticles were obtained through microwave hydrothermal synthesis. Particles with a specific surface area ranging from 51 m^2^/g to 240 m^2^/g and with sizes of 39, 29, 19, 11, 10, and 9 nm were used in the experiment. The structure of the nanomaterial was also studied by means of helium pycnometry, X-ray diffraction (XRD), and transmission-electron microscopy (TEM). The calcium-ion release into phosphate-buffered saline (PBS) was studied. The highest release of Ca^2+^ ions, i.e., 18 mg/L, was observed in HAP with a specific surface area 240 m^2^/g and an average nanoparticle size of 9 nm. A significant increase in Ca^2+^-ion release was also observed with specific surface areas of 183 m^2^/g and above, and with nanoparticle sizes of 11 nm and below. No substantial size dependence was observed for the larger particle sizes.

## 1. Introduction

Over the past years, the incidence of diseases and injuries of the skeletal system has significantly increased worldwide. These conditions are caused by the aging of the population, as well as by congenital defects, sports, traffic injuries, and other diseases [1,2,3]. The aim of tissue engineering is to accelerate bone-tissue regeneration and to enable the filling of bone defects with natural bone when bone cannot be regenerated by natural means. Treatments using biological agents, stem cells, biomimetic scaffolds, or suitable implants provide increasingly effective and reliable strategies for creating bone tissue and regenerating large defects, thus improving the quality of patients’ lives [3].

Bone tissue is composed of 60% inorganic constituents (mainly nanohydroxyapatite), 30% the organic constituent (proteins), and 10% water [4]. Natural nanohydroxyapatite contains numerous impurities in the form of potassium, magnesium, strontium, sodium, chloride, fluoride, and carbonate [4]. The second inorganic constituent of bone is whitlockite (Ca_18_Mg_2_(HPO_4_)_2_(PO_4_)_12_) [5,6]. The organic part of bone is composed mainly of type I collagen (ca. 90%) and non-collagen proteins [7,8]. Synthetic calcium phosphates, e.g., hydroxyapatite (HAP, Ca_10_(PO_4_)_6_(OH)_2_), calcium α-, and β-triphosphate (TCP, Ca_3_(PO_4_)_2_), are the subjects of continuing interest in the field of tissue engineering due to their high chemical similarity to natural hydroxyapatite [9,10,11,12]. Recent research has focused on the artificial production of nano-HAP that is as close as possible to natural HAP in terms of structure [13]. Numerous studies have confirmed the biocompatibility of nano-HAP and its usefulness in bone-tissue regeneration [14,15,16,17,18,19,20,21,22,23,24,25,26].

Calcium in the form of free ions or bound complexes plays a key role in many biological functions. The amount of calcium in the adult body is, on average, 1000 g. This element plays a key role in the mineralization of the skeleton and in other biological processes [27]. Calcium (Ca^2+^) is an intracellular messenger that controls several cellular processes, such as cell proliferation, gene transcription, and muscle contraction. The signals of Ca^2+^ involve a number of homeostatic and sensory mechanisms. These Ca^2+^ signals may induce the expression of genes that are related to bone-cell proliferation in cells [28]. In vitro studies have shown that the calcium contained in bone-regrowth scaffolds supports the increased adhesion, proliferation, and differentiation of osteoblastic MG-63 cells. Further, calcium signals promote osteoblast function through calmodulin, and through the activation of extracellular-signal-regulated kinase 1/2 (ERK1/2) and of the intracellular signaling pathway, which is important in regulating the cell cycle (PI3K/Akt pathways) [28,29,30]. In addition, calcium signals from the endoplasmic reticulum (ER) and the activation of calcineurin cause the nuclear factor of activated T cells, especially in the introduction of the IL-2 or IL-4 gene (NFAT2)’s dephosphorylation and osteoclastic gene expression [31]. The expression and control of osteoclastic genes indicates the role of calcium in bone resorption and homeostasis [31,32]. In vivo studies have shown that Ca^2+^ ions released from HA/TCP-composite scaffolds increase bone formation in rat calvarial bone defects [33]. In addition, Ca^2+^-coated titanium implants resulted in increased bone density and osteointegration in a sheep-tibial-bone model [28,30,31,32]. Thus, the control of the Ca^2+^-ion release to induce bone-tissue repair is necessary for the appropriate application of calcium-phosphate materials in tissue engineering and regenerative medicine.

The effect of particle size on particle solubility and bioavailability has been documented by many researchers [12,33,34,35,36,37,38,39,40,41,42]. This relationship is exploited mainly in pharmacy for the purpose of increasing the bioactivity of drugs [33,34,35,36,37,38]. A reduction in the size of drug particles to nanometer size increases the total effective surface area and dissolution rate. Moreover, a reduction in the particle size leads to a decrease in the thickness of the diffusion layer surrounding the drug particles, resulting in an increase in the concentration gradient [37]. Regarding hydroxyapatite, the sizes of HAP nanoparticles affect the degradation rate of this material [43]. 

The microwave hydrothermal synthesis (MHS) of hydroxyapatite nanoparticles (HAP NPs), hereinafter referred to as GoHAP, which was recently developed in our laboratory, makes it possible to control their size with nanometric precision in the range of 9 to 50 nm [13]. Its advantages include the possibility of obtaining HAP NPs that meet the requirements of purity for medical applications. This is made possible by taking advantage of microwave heating [44] and the lack of harmful by-products of the synthesis [13]. The literature [45,46,47,48,49,50,51,52,53,54,55,56,57,58,59,60,61] exhaustively describes and discusses the advantages and disadvantages of the methods of nanohydroxyapatite synthesis, e.g., for chemical precipitation, hydrothermal methods, hydrolysis, sol–gel methods, microwave irradiation, chemical vapor, the combustion technique, the pyrolysis technique, and solid-state and mechanochemical methods. If, as producers, we wish to evaluate the quality of the obtained HAP NPs for applications in bone-tissue regeneration, it is most important that the applied method makes it possible to obtain a product that is repeatable in terms of size, size distribution, shape, crystallinity, chemical purity, and phase purity [53]. 

In this paper, we take advantage of our original HAP-synthesis technology [13,62] to precisely determine the effects of GoHAP size and specific surface area on the Ca^2+^ concentration in a PBS dispersion. If the relationship between the amount of ions released, the size of the specific surface area, and the sizes of the hydroxyapatite nanoparticles is known, it is possible to program hydroxyapatite resorption [42] and create biodegradable bone grafts [53,63], or layers on titanium implants [64,65,66]. In addition, it is possible to control the degradation rate and the bone-growth-stimulation potential of biodegradable electrospun membranes coated with HAP NPs [67,68], as well as titanium implants coated with HAP NPs. The control of the resorption rate of HAP NPs may be the key to achieving a balance between the rate of material degradation and accelerated bone-tissue regeneration. This will make it possible to tailor the sizes of nanoparticles to physicians’ requirements in specific applications.

## 2. Materials and Methods

### 2.1. Materials and Synthesis of Nanoparticles

The MHS-synthesis procedure is based on the method described in [13]. Calcium hydroxide (pure Ca(OH)_2_, CHEMPUR, Piekary Śląskie, Poland) and orthophosphoric acid (85% solution H_3_PO_4_, analytically pure, CHEMPUR, Piekary Śląskie, Poland) were used for the synthesis. The synthesis was carried out in deionized water (0.06 μS/cm) purified by a water double-deionization system (HLP 20 UV deionizer, Hydrolab, Straszyn, Poland, and Ultra Toc/UV/UF, Hydrolab, Straszyn, Poland).

The GoHAP type 1 was obtained by a precipitation reaction:

10 Ca(OH)_2_ + 6 H_3_PO_4_ → Ca_10_(PO_4_)_6_(OH)_2_ + 18 H_2_O (1)

The amount of each component was adjusted to obtain calcium-deficient HAP (CDHAP) with a Ca/P ratio of 1.51. In the next step, the obtained suspension was poured into a Teflon vessel with a volume of 270 cm^3^, which was closed tightly and inserted into the high-pressure chamber of the homemade MSS2 microwave reactor in the batch mode (IHPP PAS (Warsaw, Poland), ITeE-PIB (Radom, Poland), ERTEC (Wroclaw, Poland) [69]), as described in [13]. After switching on the high-power magnetrons, microwave energy was delivered to the vessel using a waveguide. The temperature was calculated from the vapor–liquid equilibrium for water [69]. 

The power of the magnetrons was set to 3 kW. Time was counted from the moment the power was switched on. After reaching the pre-set pressure, the mean magnetron’s power was adjusted to keep the pre-selected pressure for a programmed time. Table 1 shows the values of up-heating time, total time, and pressure.

For each set of parameters, we produced 6 samples with a weight of ca. 7 g. The reaction products consisted of GoHAP particles and water only. The powders were separated from water by freeze-drying for 72 h (Lyovac GT-2, SRK Systemtechnik GmbH, Riedstadt, Germany).

### 2.2. Characterization of Nanoparticles

The powder X-ray diffraction (XRD) data were collected by the PANalytical X’Pert Pro diffractometer using monochromatic Cu Kα1 radiation and the PIXcel position-sensitive detector. The measuring range was 10–80° and the step was 0.03°. 

Scherrer’s formula was used to determine the mean crystallite size [70]. The shapes of the crystallites were considerably anisotropic, and, therefore, their lengths and widths were determined by the analysis of the XRD peak width for the 002 and 300 Bragg reflections.

The specific surface area (SSA) was examined by the Brunauer–Emmett–Teller (BET) isotherm method with the use of a Gemini 2360 surface analyzer (V 2.01, Micromeritics^®^, Norcross, GA, USA), in accordance with ISO 9277:2010 [71].

The density (DEN) was examined using a helium pycnometer (AccuPyc II 1340, Micromeritics^®^, Norcross, GA, USA), in accordance with ISO 12154:2014 [72].

Before the SSA and DEN measurements, samples were dried in a VacPrep 061 desorption station (Micromeritics^®^, Norcross, GA, USA) for a period of 2 h at 150 °C in vacuum (0.05 mbar).

The mean diameter of GoHAP, also known as the Sauter mean diameter (SMD), was calculated based on the SSA and DEN measurements using Equation (2), with the assumption of a spherical shape:

(2) $$SMD=\frac{A}{SSA\cdot 10^{18}\cdot DEN\cdot 10^{-21}}\;\left(\mathrm{nm}\right)$$

where *SMD* is the Sauter mean diameter of the nanoparticle (nm), A is the shape factor, equal to 6 for the sphere, *SSA* is the specific surface area (m^2^/g), and *DEN* is the density (g/cm^3^). This method of determining the *SMD* of GoHAP was described previously [73].

The specific-surface-area and density tests were carried out in a laboratory [74], working in accordance with PN-EN ISO/IEC 17025:2018-02 [75].

Transmission-electron microscopy (TEM) imaging was carried out using a JEOL JEM 2000EX apparatus with a beam with 200 keV of energy. Images were recorded on photographic plates and then processed into digital form using a NIKON LS-8000 ED scanner (Nikon, Tokyo, Japan). The powder samples were deposited on a 3-millimeter-diameter copper grid covered with a perforated carbon membrane, catalog symbol S147-4H, from Agar Scientific (Essex, UK). Observations were made using bright- and dark-field imaging. The size distribution of the NPs was determined by the bright field and the dark field based on the theoretical model, assuming spherical particles with a log-normal size distribution. The diameters were determined for at least 130 particles in each sample, and a histogram of the number of particles with diameters in the given range of values was created. The average particle size was calculated as an arithmetic mean using Excel software, version 2308 (Microsoft, Warsaw, Poland).

### 2.3. Determination of the Amount of Ca^2+^ Ions Released 

For this purpose, as well as to determine the chemical composition of the produced samples, inductively coupled plasma—optical emission spectrometry (ICP-OES) with induction in argon plasma (iCAP 6000series, Thermo Scientfic, Cambridge, United Kingdom) was used. The samples for the tests were prepared as follows.

The nanopowders were dried for 12 h at (100 ± 2 °C). Next, 0.1 g of each type of powder was weighed and placed in plastic containers with a volume of 50 mL. Subsequently, 20 mL of the phosphate-buffered saline (PBS) solution, pH = 7.4 ± 0.1 (Sigma Aldrich, Saint Louis, MS, USA), was added to the containers using a pipette. The sealed plastic containers were placed in a water bath (Heating Bath B-491, BUCHI, Flawil, Switzerland) at 37 ± 1 °C. The batch was shaken in the longitudinal motion at 2 Hz. Ion-concentration analyses using the ICP-OES technique were carried out on the following days: 1, 3, 7, 9, 14. For each time point, analyses of 2 samples of each type were carried out.

The PBS samples for the ICP-OES tests were collected by filtering the particles from the suspension.

To determine the chemical composition, 0.2 g each of GoHAP type 1–type 6 was collected from the containers and transferred to the Teflon (polytetrafluoroethylene) container of the microwave mineralizer (Magnum II, Ertec, Wroclaw, Poland). Next, 20 mL of HCL and 4 mL of HNO_3_ were added. After 10 min of treatment at a power of 800 W, the powder sample was dissolved. An ICP analysis was then carried out to determine the content and ratio of Ca^2+^, PO_4_^3−^ ions, and trace elemental impurities: Mg, Si, Al, Fe, Na, Mn. The procedure was repeated for each powder sample.

An analysis of pH and conductivity of the PBS buffer was performed using a pH meter (SevenExcellennce, Multiparameter, Mettler Toledo, Greifensee, Switzerland). An InLab Expert Pro-ISM pH electrode with a built-in temperature sensor was mounted to the instrument to measure pH, and a conductivity probe, InLab 731-ISM Cond (Mettler Toledo, Greifensee, Switzerland), was used to measure conductivity. The temperature of the buffers was maintained at 35 °C using an electric heater (babyono, Natural Nursing, Poznan, Poland). The pH electrode was calibrated with Mettler Toledo technical buffers at three pH points: 4.03, 7.00, and 9.01, respectively. Measurements were made by immersing the electrode in the PBS solutions, which were prepared in the same manner as for the ICP-OES measurements. After each measurement, the electrode was rinsed with deionized water with a conductivity of 0.06 μS/cm and treated in a double deionization system (HLP 20 UV, Hydrolab, Straszyn, Poland, and Ultra Toc/UV/UF, Hydrolab, Straszyn, Poland).

## 3. Results

### 3.1. Synthesis of Nanoparticles

**Figure 1:**
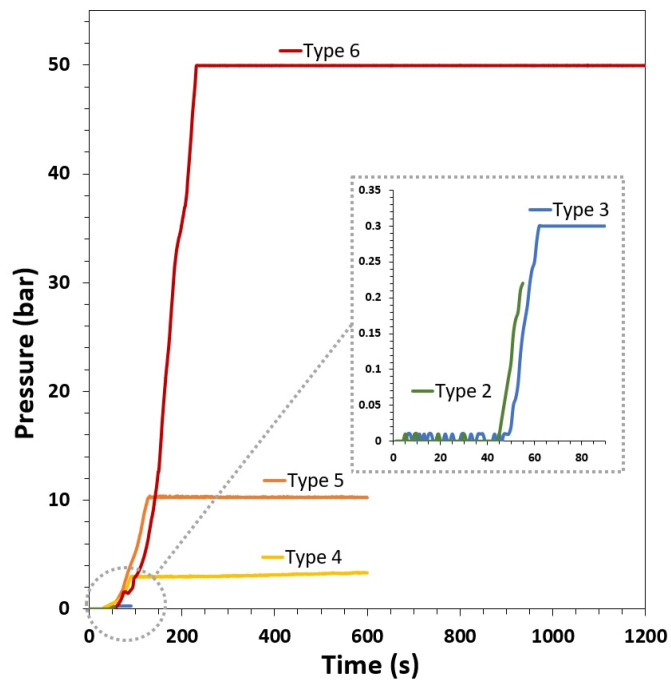
Pressure–time plots for the GoHAP synthesis of size-controlled nanoparticles (GoHAP Type 2–Type 6). Time is counted from the moment the magnetron was switched on and the start of power delivery.

The MSS2 reactor allows the rapid heating of reactants, a rapid pressure increase, fast cooling, and, thus, excellent control of the reaction time [69]. Figure 1 shows the pressure–time plots for the GoHAP synthesis. Table 1 lists the produced GoHAP types depending on the process parameters. Time was counted from the moment the magnetron was switched on and the start of the power delivery. The inset in Figure 1 reveals that there was a delay of ca. 43 s between the moment power was switched on to the moment when the pressure sensors of the reactor registered an increase in pressure. Thus, after 43 s, the pressure started to exceed the atmospheric pressure, indicating that the temperature exceeded 100 °C. The heating rate was in the range of 1–2 °C/s.

The operation of the magnetrons was switched from full power to the pulsed mode so that the mean power could be adjusted to keep a constant pressure after the heating time. This allowed the nanoparticles to grow. For GoHAP type 2, the heating was interrupted after 55 s and the power was switched off. The heating time was 60 s for the GoHAP type 3. To shorten the time needed to produce larger particles, the pressure and temperature were raised. The particle-growth phase was 500 s for the GoHAP type 4, 480 s for the GoHAP type 5, and 1000 s for the GoHAP type 6. The unique characteristics of the microwave hydrothermal technology using the MSS2 reactor in terms of time and pressure control are evident.

### 3.2. Characterization of Nanoparticles

The characteristics of the GoHAP nanomaterial—the specific surface area, density, and particle size of the produced GoHAP hydroxyapatite—are presented in Table 2 and Table 3. When combining information from Table 1 and Table 3, it can be seen that an increase in the process pressure and temperature or time leads to an increase in the particle size. 

**Figure 2:**
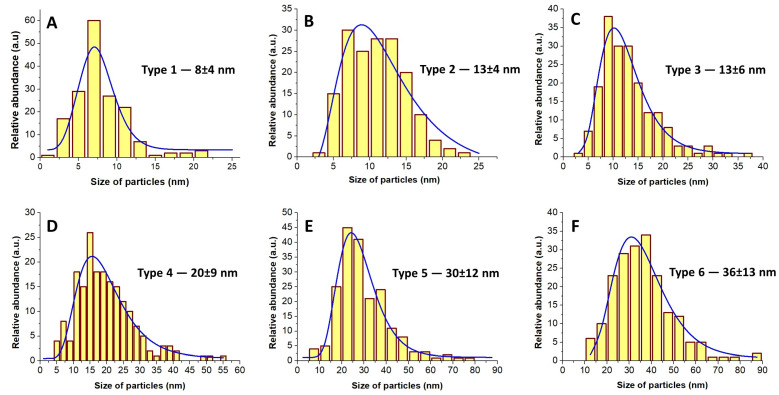
The histogram of the particle-size distribution of GoHAP samples (TEM method): (**A**) Type 1, (**B**) Type 2, (**C**) Type 3, (**D**) Type 4, (**E**) Type 5, (**F**) Type 6.

Figure 2 presents the particle-size distributions of the GoHAP particles, which were obtained from the analysis of the TEM images. In line with the increase in the synthesis temperature and in the synthesis time, the increase in the average particle size and in the size distribution is visible. One should note the results of the sizes of the Type 2 and Type 3 samples: despite the identical average particle size (13 nm), these samples had different particle-size distributions.

**Figure 3:**
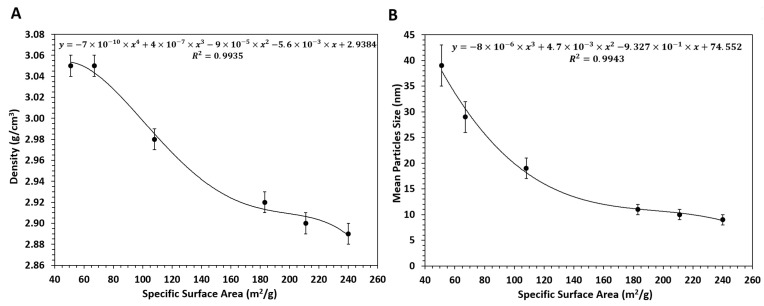
Density as a function of specific surface area (**A**), and mean particle size as a function of specific surface area (**B**). Experimental and calculated correlation of density as a function of mean particle size based on BET for GoHAP Type 1–Type 6.

Figure 3 shows a correlation between the density and the specific surface area. The greater the mean particle size (and, thus, the smaller the specific surface area), the greater the density.

**Figure 4:**
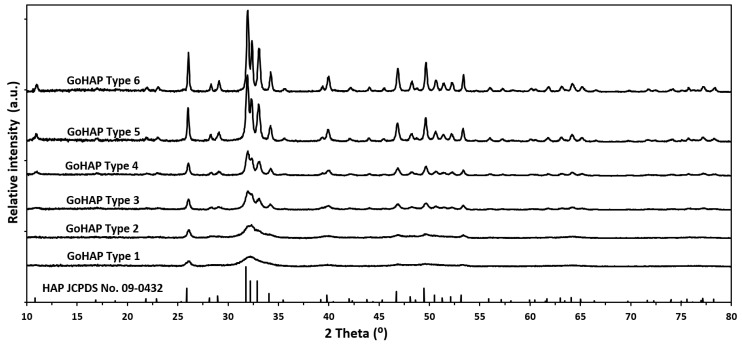
X-ray-diffraction-line profiles of GoHAP samples.

Figure 4 shows X-ray-diffraction spectra for all the GoHAP nanoparticles studied. The diffraction peaks correspond to pure hydroxyapatite. The decrease in the width of the diffraction peaks indicates the increasing mean size of the GoHAP nanoparticles.

Table 4 shows the Ca/P ratio for the GoHAP type 1–type 6 nanoparticles. The ICP-OES analysis showed that they consisted of calcium and phosphorus, in a ratio of 1.52 ± 0.01. In addition, minor contamination with Mg, Si, Al, Fe, Na, and Mg was observed (Table 5). The ratio of calcium to phosphorus indicates that the produced nanoparticles represent calcium-deficient hydroxyapatite (CDHA). The ion-substituted CDHA has Na^+^, K^+^, Mg^2+^, Sr^2+^ for Ca^2+^, CO_3_^2−^ for PO_4_^3−^ or HPO_4_^2−^, and F^−^, Cl^−^, CO_3_^2−^ for OH^-^, and with water it forms biological apatite —the main inorganic part of animal and human bone in normal and pathological calcifications [76,77].

The total impurity content was 0.40 ± 0.06 wt.%. The differences in impurity content between the GoHAP types were less than 15% of the total impurity content.

**Figure 5:**
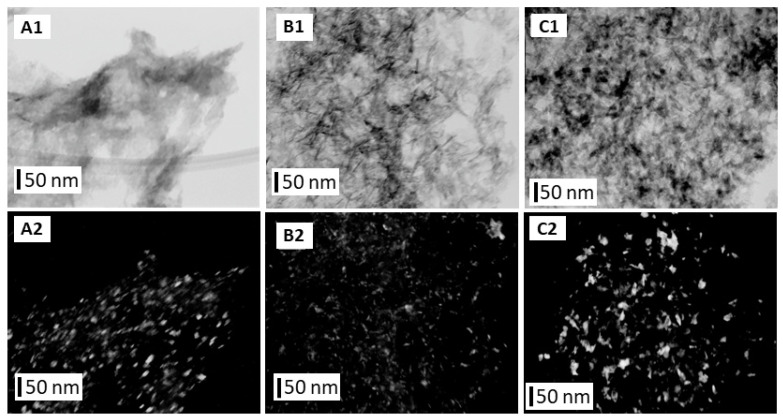
TEM images of GoHAP samples: (**A1**,**A2**) type 1, (**B1**,**B2**) type 2, (**C1**,**C2**) type 3. TEM (**A1**–**C1**) TEM images obtained using bright-field imaging. (**A2**–**C2**) TEM images obtained using dark-field imaging.

**Figure 6:**
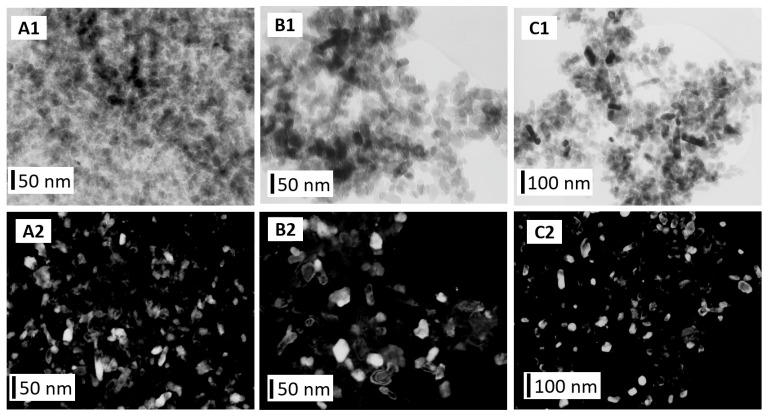
TEM images of GoHAP samples: (**A1**,**A2**) type 4, (**B1**,**B2**) type 5, (**C1**,**C2**) type 6. (**A1**–**C1**) TEM images obtained using bright-field imaging. (**A2**–**C2**) TEM images obtained using dark-field imaging.

The TEM images (Figure 5 and Figure 6) obtained using the bright- and dark-field techniques showed differences between the shapes and sizes of the GoHAP type 1–type 6 nanoparticles. The GoHAP type 1 nanoparticles had the smallest sizes and a needle-like shapes. A very similar shape was obtained for the GoHAP Type 2 particles. As the nanoparticles grew, they took on increasingly spherical shapes (GoHAP type 3–type 6).

### 3.3. Calcium-Ion-Release Results

**Figure 7:**
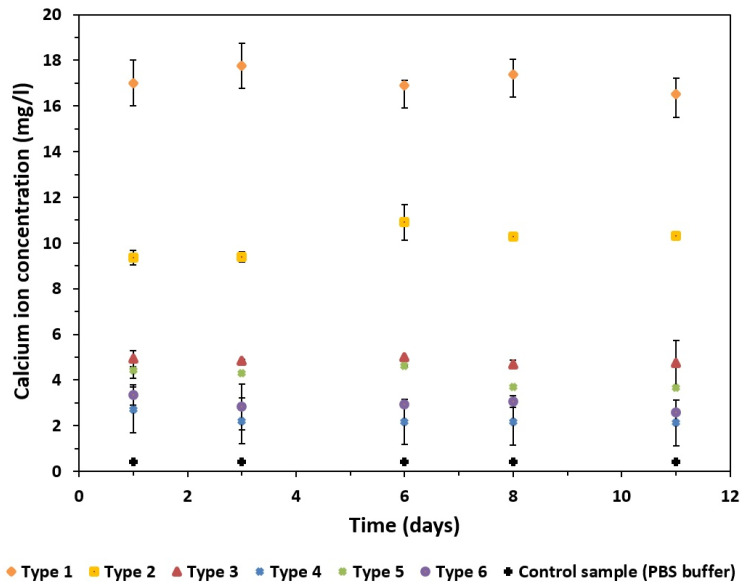
Concentrations of calcium ions released from GoHAP Type 1–Type 6.

This study showed a strong effect of the specific surface area of GoHAP on the amount of ions released (Figure 7). The release of calcium ions at the highest concentrations, i.e., ca. 18 mg/L, was observed for the GoHAP type 1 with a specific surface area of 240 m^2^/g and an average nanoparticle size of 9 nm. As the nanoparticle size increased and the specific surface area decreased, the amount of calcium ions released into the buffer solution decreased. Compared to the GoHAP type 1 (SSA 240 m^2^/g), a significant decrease in solubility and in the associated calcium-ion release was already observed for the slightly larger GoHAP type 2 nanoparticles, with an average nanoparticle size of 10 nm (183 m^2^/g), and the GoHAP type 3, with an average nanoparticle size of 11 nm (108 m^2^/g). The decrease in the amount of released Ca^2+^ ions was ca. ↓7 mg/L, with a difference in the developed specific surface area of 30 m^2^ /g (GoHAP type 2) and ca. ↓12 mg/L, and with a difference in the developed specific surface area of 57 m^2^/g (GoHAP type 3) compared to the GoHAP type 1. Small amounts of Ca^2+^-ion release, ranging from ca. 2.3 mg/L to 4.1 mg/L, were observed for the GoHAP type 4–6 nanoparticles. Therefore, it seems that there is a threshold nanoparticle size at 11 nm and a threshold SSA at 108 m^2^/g, above which Ca^2+^-ion release becomes size-independent. 

In each of the GoHAP types studied, the Ca^2+^-ion concentration stabilized after 1 day in the buffer solution (Figure 7). After this time, nearly constant levels of calcium-ion release were observed, which indicates that an equilibrium state was achieved.

**Figure 8:**
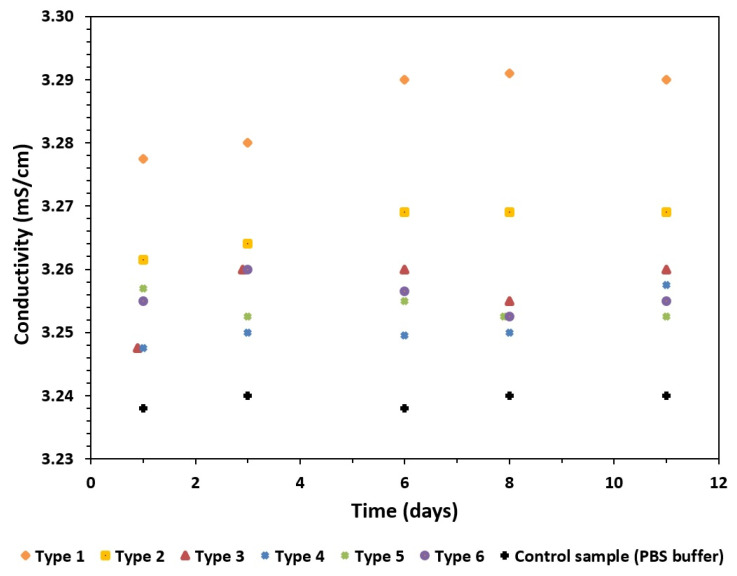
Conductivity of PBS filtrate during dissolution tests of GoHAP type 1–type 6.

**Figure 9:**
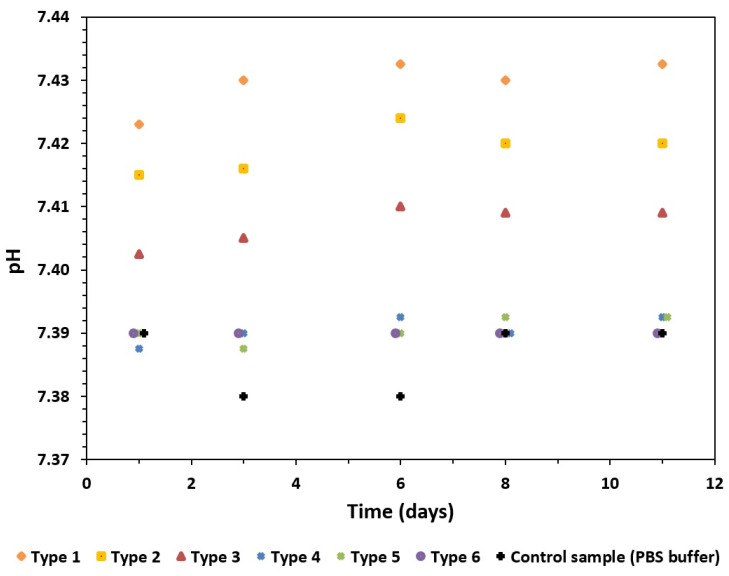
The PH of PBS filtrate during the degradation test of GoHAP type 1–type 6.

The changes in the conductivity and pH of the solution were correlated with the amount of calcium ions released (Figure 8 and Figure 9). The largest increase in conductivity was observed when the GoHAP type 1 nanoparticles with the smallest sizes and the highest specific surface areas were dissolved (Figure 8). The increase in conductivity decreased as the specific surface area of the GoHAP decreased.

During the calcium-ion release tests, the pH increased steadily, depending on the development of the specific surface area, and maintained the following relationship: highest specific surface area—highest pH; lowest development of specific surface area of nanoparticles—lowest pH (Figure 9). However, the differences in the pH values of the solutions during the solubility testing of the GoHAP type 1 (240 m^2^/g) and type 6 (51 m^2^/g) nanoparticles were small and accounted for about 0.05 points on the pH scale.

## 4. Discussion

### 4.1. Structural and Chemical Characterization

The studies of the GoHAP nanostructure confirmed a gradual increase in the particle size in line with the increase in time and in pressure and temperature of the microwave synthesis. In addition, the particle shape transformed gradually from a plate or a needle to an ellipsoid. The possible mechanism underlying the change in the shapes of GoHAP particles was described in the paper by Kozerozhets et al. [78]. Furthermore, the pycnometric density of the particles gradually increased in line with the time and with the pressure and temperature of the synthesis. The obtained GoHAP nanoparticle samples had a lower density than the theoretical density of hydroxyapatite, which is 3.15 g/cm^3^ [79,80]. This correlation was previously found for zirconia (ZrO_2_) nanoparticles [81] and doped and undoped zinc oxide (ZnO) nanoparticles [82,83,84]. It is attributed to the effect of the surfaces of nanoparticles on their mean density. Even nanoparticles with the maximum possible degree of crystallinity have dangling bonds on their surfaces, to which -OH groups in oxides may attach [81]. In addition to the presence of hydroxides, another reason for the density–size correlation is that the thickness of the amorphous phase on the nanoparticle surface decreases as the size of the nanoparticles increases [83]. In a pycnometer study [85,86], such surfaces contributed to the volume occupied by the nanoparticle, so that the mean density measured decreased as the specific surface area increased.

The hydroxyapatite unit cell (both synthetic and natural) usually displays a hexagon crystal system, with a P63/m space group [87,88], even if the occurrence of monoclinic HAP is well-known [89]. The crystal structure of the GoHAP type 1 sample was identical to that of human bone [90], while the crystal structure of the GoHAP type 6 sample was identical to that of human tooth enamel [13]. The XRD data show that the particles had non-spherical shapes. The smallest, type 1 and type 2, had platelet shapes, with the larger surface parallel to the (100) planes. The aspect ratio varied for the particles with SSA values equal to or above 183 m^2^/g in the range of 1.7–4.2.

The differences between the sizes, as measured by means of the XRD method and calculated from the SSA, were not significant, because the SSA delivered a mean value, while the XRD data depended on the axis selected for the analysis. Further, in the case of SSA, the smallest particles may have delivered the highest contribution to the surface area, while in the case of XRD studies, they may have disappeared into the background due to very broad peaks.

When comparing the results of the crystallite sizes calculated from the XRD data with the results of the average particle sizes calculated from the TEM images, it was found that they were virtually identical or fell within the standard deviation. This means that monocrystalline hydroxyapatite was obtained in all the samples (one particle was built of one crystallite). The results of the TEM imaging are consistent with those of the XRD analysis—for the smallest particles, especially type 1 and type 2, the shape was platelet- or needle-like, while for the larger particles, the shape was close to spherical.

The structural characterizations showed that the MHS method permits the production of a high-purity nanomaterial with a constant chemical composition as a function of size. The density increases in line with the increase in size, due to the decreasing fraction of atoms situated on the surface. The smallest particles, with SSA values below 183 m^2^/g, have a highly non-equilibrated elongated shape, which is characteristic of particles formed in a short time. For longer times or higher temperatures, the aspect ratio of the nanoparticles decreases, tending towards greater sphericity.

Taking all these results into consideration, it is justified to regard SSA as the main characteristic parameter of the nanomaterial, on which all other properties, size, shape, and density, depend.

Regarding the chemical composition, no SSA effect was detected. The calcium/phosphorus ratio for calcium-deficient hydroxyapatite (CDHA) (Ca_10x_(HPO)_4x_(PO)_46−x_(OH)_2−x_ (0 < x < 1) is 1.5–1.67. As shown in Table 3, x = 1.52, the calcium/phosphorus ratio corresponds to human HAP. A calcium-deficient composition was selected specifically for this study, so that the synthetic GoHAP would mimic the natural nano-HAP as much as possible. 

The total impurity content, as shown in Table 4, was 0.40 ± 0.06 wt.%. The differences in the impurity content between the various GoHAP types were less than 15% of the total impurity content.

### 4.2. Ion-Release Study

For standard commercial hydroxyapatite with particle sizes much larger than those of nanomaterials, the most important parameters are the molar Ca/P ratio, basicity/acidity, and solubility. The lower the Ca/P molar ratio, the more acidic the powder and the more water-soluble the calcium orthophosphate [76,77]. In this respect, hydroxyapatite is one of the most stable calcium phosphates.

**Figure 10:**
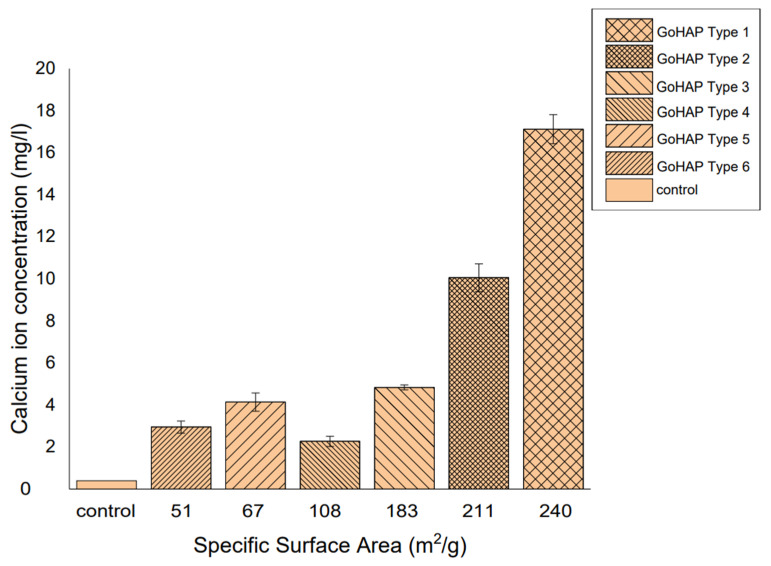
Correlation between the amount of calcium ions released and the specific surface area of GoHAP type 1–type 6.

However, as the present study shows, a reduction in the sizes of HAP nanoparticles results in active nanoparticles that release calcium ions, i.e., the stability of the particles decreases. They are therefore bioactive and potentially biodegradable. For nanoparticles smaller than 39 nm immersed in PBS, calcium-ion release is observed, but solubility increases significantly for a threshold size lower than 11 nm. The greatest differences in nanoparticle solubility were observed in the nanoparticles with specific surface areas in the range of 240–183 m^2^/g (Figure 10).

**Figure 11:**
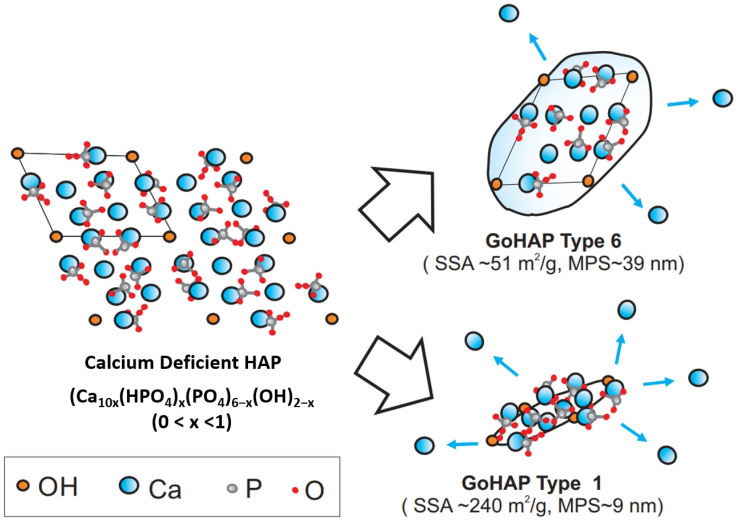
Illustration of the effect of the specific surface areas of hydroxyapatite nanoparticles on calcium-ion release. SSA—specific surface area; MPS—mean particle size.

Such properties are sought and explored in the field of nanotechnology, in which the chemical composition of the material is constant, but the size of the particles or the crystallites of the material are variable. This was the case in the present paper. The specific surface area is defined as the external surface area of a substance per unit mass of that substance. This ratio depends on the parameters of size and shape; the smaller the solid and the more needle-like its shape, the greater the specific surface area. In nanoparticles with dimensions of several nm, large fractions of atoms are situated on the surface and, therefore, their free energy is high compared to large particles [91,92] (Figure 11).

The concentration of Ca^2+^ ions in PBS depends on the SSA value, and not on time. This effect can be explained in terms of an equilibrium between the liquid phase and the specific surface of the solid phase, rather than its mass. This effect is in line with the treatment of the specific surface area (per unit volume or per unit weight) as an independent thermodynamic variable. We regard SSA as the crucial parameter. This is because the sizes of nanoparticles are difficult to determine both with microscopy methods and with XRD methods, especially for complicated shapes, and when there is a size distribution. A focus on the specific surface area as a key parameter is described in multiple papers [93,94]. The effect of the specific surface area’s value (above 183 m^2^/g) on the solubility of HAP nanoparticles, which we observed, was explained in the paper by Fu et al. [95]. It should be noted that Fu et al. [95] assumed spherically shaped nanocrystals for the purpose of their calculations. The calculated results of the thermodynamic properties of the surfaces showed that the limiting size (diameter) of the nanocrystals was 20 nm. When the size was less than 20 nm, the effect of the particle size on the thermodynamic properties of the surface increased and deviated from linear variation. Spherical HAP nanoparticles measuring 20 nm have specific surface areas of ca. 100 m^2^/g and the assumption of a diameter of less than 20 nm (specific surface area above 100 m^2^/g) was confirmed by our four samples, i.e., from GoHAP type 1 (240 m^2^/g) to Go HAP type 4 (108 m^2^/g). Fu et al. [95] also discovered that an important factor, in addition to the specific surface area value itself, in the thermodynamic properties is the shape of the nanocrystals. With an identical equivalent diameter of particles, the more the shape deviates from sphere, the stronger the thermodynamic properties of the surface (absolute value) [95]. The shape-criterion and the specific-surface-area values were displayed only by the GoHAP type 1 and GoHAP type 2 samples, in which we observed significantly increased hydroxyapatite solubility. To the best of our knowledge, our study is the first to report the effect of the specific surface area’s value on nanohydroxyapatite solubility [39,96,97,98,99,100,101,102,103,104,105,106,107,108,109,110,111,112,113,114,115,116]. It must be underlined that the novelty of our paper in relation to the papers reported previously [39,96,97,98,99,100,101,102,103,104,105,106,107,108,109,110,111,112,113,114,115,116] is in the values of the specific surface area of the HAP-NP samples that we used in the solubility tests (from 51 m^2^/g to 240 m^2^/g). A good comparative example is the paper by Tang et al. [39], who examined the size effects in the dissolution of hydroxyapatite for HAP-NP samples with specific surface areas of 24.2 m^2^/g, 32.4 m^2^/g, and 55.1 m^2^/g. The dissolution studies the authors carried out for the different undersaturations lasted only 100 min. Our tests lasted 11 days and referred to the state of an excess amount of solid HAP NPs in the suspension relative to the solubility product (which was significantly above the state of equilibrium). Tang et al. [39] discovered that in unsaturated biological environments, there is a metastable HAP phase that depends on the effects of particle sizes, resulting in the self-inhibition of dissolution, or even the suppression of the dissolution reaction.

If the ion concentration, the volume of the PBS, and the weights of the nanoparticles are known, it is possible to calculate the amount of dissolved GoHAP. For the highest Ca^2+^-ion concentration, 18 mg/l, the weight of the dissolved hydroxyapatite was 0.9 mg, i.e., 0.9% of the sample. This is a considerable amount, which dissolved in just one day in the PBS. It is plausible that for each sample, the smallest particles underwent dissolution. In the further studies, we will examine the effect of storage in the solution on the nanostructure of the particles.

Further, an effect of the surface development on the pH and the conductivity of the solution was observed. The changes in the pH and conductivity ranged from ca. 3.24 to ca. 3.29 with an increase in the SSA value. Although the calcium-ion concentration stabilized after one day, the conductivity values stabilized after three days. However, the conductivity can hardly be correlated with calcium release only, as it depends on the overall composition of the solution, with a range of ions present.

Regarding the practical implications of this study for the development of nano-HAP as a material to enhance bone regeneration, there are two contradictory trends to be considered. On one hand, the larger the specific surface area, the greater the activity of the particles in the ion release and, possibly, in the biodegradation. On the other hand, the thermodynamic stability of these particles is limited, as a very high SSA value relates to high energy per unit weight or volume. This may limit the application of these materials because the shelf time would be short. It seems, from the present study, that the optimal SSA value is between 180 m^2^/g and 200 m^2^/g. For these values, the aspect ratio of the particles decreases to a stable level, so that the shape of the particles does not change in a significant way with time and possible further SSA decreases. On the other hand, the calcium-ion release remains at a high level. The MHS technology makes it possible to tune the particle size in this narrow gap of values.

Appropriate calcium-phosphate homeostasis is essential for normal bone function. In cases of large bone defects, insufficient calcium-ion release can contribute to a lack of or very slow bone-tissue regeneration. On the other hand, excessive calcium release can lead to undesirable tissue calcification. Therefore, it is necessary to find the optimal amount of calcium at which the calcium signal intensifies the induction of gene expression toward bone cells and thereby accelerates bone-tissue regeneration, which will be the subject of our future work.

## 5. Conclusions

Hydroxyapatite nanoparticles with the following average sizes were obtained with the use of the original method of microwave hydrothermal synthesis: 39 nm (51 m^2^/g), 29 nm (67 m^2^/g), 19 nm (108 m^2^/g), 11 nm (183 m^2^/g), 10 nm (211 m^2^/g), and 9 nm (240 m^2^/g). By varying the temperature and synthesis time, microwave hydrothermal synthesis makes it possible to precisely tune the specific surface area, shape, and density of hydroxyapatite nanoparticles, while keeping their chemical composition constant. A threshold specific surface area of 183 m^2^/g (11 nm) was found; above this threshold, the solubility of hydroxyapatite nanoparticles in phosphate-buffered saline increases significantly. Particles with optimal properties for application as bone-graft materials should have a specific surface area value in the range of 180–200 m^2^/g. The calcium release from the nanoparticles immersed in phosphate-buffered saline increased strongly above this specific surface area value. This effect can be exploited to produce bioactive hydroxyapatite. The nanoparticle size is therefore crucial when designing materials for bone-tissue regeneration. 

## Figures and Tables

**Table 1:** Microwave-synthesis parameters.

GoHAP	Heating Time (s)	Total Reaction Time (s)	Pressure (bar)	Temperature (°C)
Type 1	-	-	-	-
Type 2	55	55	0.2	115
Type 3	60	90	0.3	125
Type 4	100	600	3	130
Type 5	120	600	10	175
Type 6	200	1200	50	260

**Table 2:** GoHAP characterization. Standard deviation is given for each value (±).

GoHAP	Specific Surface Area, a_s_ (m^2^/g)	Skeleton Density, ρ_s_ ± SD (g/cm^3^)
Type 1	240	2.89 ± 0.01
Type 2	211	2.90 ± 0.01
Type 3	183	2.92 ± 0.01
Type 4	108	2.98 ± 0.01
Type 5	67	3.05 ± 0.01
Type 6	51	3.05 ± 0.01

**Table 3:** Comparison of the GoHAP NP sizes with different methods. D_(002)_—crystallite size for crystal plane 002. D_(300)_—crystallite size for crystal plane 300. Standard deviation is given for each value (±). d—mean particle size (diameter); SD—standard deviation; SSA—specific surface area; TEM—transmission-electron microscopy.

GoHAP^TM^	Mean Particle Size Based on TEM Method, d_TEM_ ± SD (nm)	Mean Particle Size Based on SSA, d_SSA_ ± SD (nm)	Mean Size of Crystallites Based on Scherrer’s Formula		
			Length D_(002)_ ± SD (nm)	Width D_(300)_ ± SD (nm)	Aspect Ratio (D_(002)_/D_(300)_)
Type 1	8 ± 4	9 ± 1	14 ± 7	5 ± 1	2.8
Type 2	13 ± 4	10 ± 1	21 ± 12	5 ± 2	4.2
Type 3	13 ± 6	11 ± 1	29 ± 15	17 ± 7	1.7
Type 4	20 ± 9	19 ± 2	33 ± 17	23 ± 8	1.4
Type 5	30 ± 12	29 ± 3	43 ± 20	27 ± 10	1.6
Type 6	36 ± 13	39 ± 4	51 ± 24	32 ± 11	1.6

**Table 4:** Calcium-to-phosphate molar ratio of GoHAP Type 1–Type 6. Data obtained from ICP-OES analyses.

GoHAP	Calcium (mol)	Phosphorus (mol)	Calcium-Phosphorus (Ca/P) Ratio
Type 1	8.29	5.44	1.52
Type 2	8.09	5.36	1.51
Type 3	9.67	6.40	1.51
Type 4	8.71	5.74	1.52
Type 5	5.38	3.54	1.52
Type 6	9.29	6.09	1.53

**Table 5:** Impurity content of GoHAP type 1–type 6. Data obtained from ICP-OES analyses.

GoHAP	Magnesium (wt%)	Silicon (wt%)	Aluminum (wt%)	Iron (wt%)	Sodium (wt%)	Sodium (wt%)
Type 1	0.225	0.053	0.019	0.021	0.082	0.0009
Type 2	0.219	0.056	0.018	0.018	0.083	0.0009
Type 3	0.258	0.049	0.019	0.020	0.074	0.010
Type 4	0.232	0.054	0.019	0.020	0.087	0.010
Type 5	0.144	0.043	0.013	0.014	0.073	0.006
Type 6	0.252	0.050	0.019	0.026	0.083	0.010
Mean value	0.222 ± 0.041	0.051 ± 0.005	0.018 ± 0.002	0.020 ± 0.004	0.080 ± 0.006	0.006 ± 0.004

## Data Availability

Not applicable.
